# Tailored PVA/ECM Scaffolds for Cartilage Regeneration

**DOI:** 10.1155/2014/762189

**Published:** 2014-07-24

**Authors:** Elena Stocco, Silvia Barbon, Daniele Dalzoppo, Silvano Lora, Leonardo Sartore, Marcella Folin, Pier Paolo Parnigotto, Claudio Grandi

**Affiliations:** ^1^Department of Pharmaceutical and Pharmacological Sciences, University of Padua, Via Marzolo 5, 35131 Padua, Italy; ^2^Foundation for Biology and Regenerative Medicine, Tissue Engineering and Signaling (TES) ONLUS, Via De Sanctis 10, Caselle di Selvazzano Dentro, 35030 Padua, Italy; ^3^Operative Unit of Plastic Surgery, Sant'Anna Hospital, Via Ravona 20, 22020 Como, Italy; ^4^Department of Biology, University of Padua, Viale Colombo 3, 35121 Padua, Italy

## Abstract

Articular cartilage lesions are a particular challenge for regenerative medicine due to cartilage low self-ability repair in case of damage. Hence, a significant goal of musculoskeletal tissue engineering is the development of suitable structures in virtue of their matrix composition and biomechanical properties. The objective of our study was to design *in vitro* a supporting structure for autologous chondrocyte growth. We realized a biohybrid composite scaffold combining a novel and nonspecific extracellular matrix (ECM), which is decellularized Wharton's jelly ECM, with the biomechanical properties of the synthetic hydrogel polyvinyl alcohol (PVA). Wharton's jelly ECM was tested for its ability in promoting scaffold colonization by chondrocytes and compared with polyvinyl alcohol itself and the more specific decellularized cartilage matrix. Our preliminary evidences highlighted the chance of using Wharton's jelly ECM in combination with PVA hydrogels as an innovative and easily available scaffold for cartilage restoration.

## 1. Introduction

Cartilage degeneration, due to congenital abnormalities or disease and trauma, represents a major health problem of great clinical consequence [1, 2]. In case of damage, cartilage is not capable of healing as it is an avascular and aneural tissue; moreover, its cellular components, chondrocytes, have low mitotic ability [3, 4]. Cartilage lesions are generally believed to progress to severe forms of osteoarthritis [5, 6], leading to pathologic changes in the joints with consequent pain, inflammation, and functional disability [7, 8]. Injuries which reach the subchondral bone may induce a systemic reaction and generate reparative tissue. Although type II collagen may be produced by this reparative tissue, it consists predominantly of type I collagen, resulting in the formation of fibrocartilage which does not have the biomechanical properties of articular cartilage [9].

The poor regenerative potential of cartilage and the unsatisfactory current clinical therapies have led to the research of strategies providing solutions to the treatment of focal defects [10, 11]. An emerging and promising field for the generation of tissue substitutes is tissue engineering. The basic approach to tissue engineering depends upon the interaction between cells, scaffolds, and signalling factors to create* in vitro* a biological tissue construct to implant* in vivo* mimicking the tissue of interest; engineering cartilage is no exception to this approach [1, 12, 13].

Implanting the patient's own chondrocytes into the cartilage defect is a method called “matrix-associated autologous chondrocyte transplantation” (MACT): it is performed with either natural or synthetic polymer-based scaffolds [14]. Amongst synthetic biomaterials, hydrogels have demonstrated their ability to simulate human tissue better than any other class. In particular, physically cross-linked poly(vinyl alcohol) (PVA) hydrogels are attractive tools in cartilage tissue engineering as they have a viscoelastic behaviour comparable with that of articular and meniscal cartilage. PVA hydrogels are physically cross-linked through freeze-thaw (FT) cycles: exposing the polymer solution to cold temperatures, water freezes and PVA is expelled forming areas of high PVA concentration. PVA chains come into close contact with each other and crystallite formation as well as hydrogen bonding occurs. These interactions remain intact after thawing and create a nondegradable 3D hydrogel network. It is possible to tailor mechanical properties of the hydrogel acting on the number of FT cycles [15]. However, despite PVA biocompatibility, its low protein adsorption property results in low cell adhesion compared with other hydrogels [3].

In the body, cells are embedded in the extracellular matrix (ECM) which is made up of protein fibres interwoven in a network of glycosaminoglycan (GAG) chains. The ECM influences cellular responses like survival, development, and behaviour by interacting with cellular adhesion molecules, growth factors, binding proteins, proteolytic enzymes, and enzyme inhibitors [16]. Hence, ECM has been successfully used as a scaffold for constructive remodelling of multiple tissues in both preclinical studies and in human clinical applications [17]. However, despite ECM, derived scaffolds offer promising regenerative responses in many settings; in some applications, more robust and long lasting mechanical properties are necessary [18]. A composite scaffold, strong and bioactive, may represent an interesting solution to this problem. In this work, we have investigated how to realize a scaffold able to sustain articular cartilage regeneration. We have combined mechanical properties of PVA and bioactive ones of ECM. In particular, our attention focused on the investigation of an alternative ECM tissue derived from the umbilical cord Wharton's jelly in comparison with the more specific cartilage matrix.

## 2. Materials and Methods

### 2.1. Culture Media and Reagents

All chemicals and reagents were obtained from Sigma-Aldrich Chemical Company (St. Louis, MO, USA), except for phosphate-buffered saline (PBS) tablets, Dulbecco's modified Eagle's medium/F12 (DMEM/F12) (2 : 1) (Gibco Invitrogen Corporation, Paisley, UK), sodium chloride (Fluka, Basel, Switzerland), the Vectashield Mounting medium for fluorescence with DAPI (Vector Laboratories, Burlingame, CA, USA), Movat pentachromic staining kit (Diapath, Bergamo, Italy), Masson trichrome staining kit (Bio-Optica, Milano, Italy), and collagenase B (Roche, Basel, Switzerland). MilliQ grade water was prepared with a MilliQ Academic system (Millipore, Bedford, MA, USA).

### 2.2. Scaffold Manufacture

Three different scaffold groups were investigated to analyse their ability in sustaining chondrocytes adhesion and proliferation: the PVA hydrogel alone and the PVA hydrogel combined with Wharton's jelly (W's J) derived matrix; the PVA hydrogel combined with articular cartilage (AC) derived matrix.

For the first group, an aqueous solution of 16 wt% PVA (Mw 146,000–186,000 Da, 99+% hydrolysed) was prepared by heating the polymer suspension for 48 hours at 90°C, under stirring, until complete dissolution. The PVA solution was then slowly cooled down to room temperature. Finally, a volume of 0.7 mL of the PVA solution was cast into each well of a 24-well tissue culture plate (mould) (BD Falcon, Franklin Lakes, NJ, USA).

For composite scaffolds, ECMs were gained from umbilical cord and cartilage samples collected after obtaining informed consent of donors. All tissue samples were rinsed several times in PBS containing 2% penicillin/streptomycin solution in order to remove any residual blood.

After taking off blood vessels from umbilical cords, Wharton's jelly and cartilage were minced into small fragments that were all gathered in a 50 mL tube (BD Falcon). Fragments were then decellularized according to the detergent-enzymatic method by Meezan and collaborators [19]. Briefly, samples were soaked in distilled water for 72 h at 4°C, changing the aqueous solution every 2 h, 4% sodium deoxycholate for 4 h at room temperature (RT), and 2,000 KU (Kunitz Units) DNase-I in 1 M NaCl for 2 h at RT. After decellularization, 1 g of W's J or cartilage was soaked with 15 mL of 10% acetic acid solution (2.5 M) in deionized water (dH_2_O) and homogenized at 0°C using Ultra-Turrax homogenizer (Janke & Kunkel GmbH, Staufen, Germany) 8 times/20 sec with intervals of 5 min. This stage was led in an ice bath. For total protein quantitation, 1 mL of each homogenate was analysed as described in Section 2.5. In parallel, 400 *μ*L of matrix solution was cast into each well of a 24-well cell culture plate (mould) and frozen at −20°C before being lyophilized overnight using an under-vacuum evaporator (Speed Vac Concentrator Savant, Instruments Inc., Farmingdale, NJ, USA). Composite scaffolds of PVA and ECM were prepared setting down carefully a thin matrix layer upon PVA solution poured in 24-well plates. A freeze-thaw treatment was used to physically cross-link the hydrogel and to embed the lyophilized matrix upon it. Briefly, the coated plate was frozen at −20°C and slowly thawed at −2.5°C for 5 times. At the end of the freeze-thawing treatment, composite scaffolds were kept at −20°C until use.

### 2.3. Mechanical Testing of PVA Hydrogels

Hydrated 16 and 25 wt% PVA hydrogels underwent tensile tests. Analyses were performed in a universal testing machine Bose (Electroforce, Eden Prairie, MN, USA), at RT and with a crosshead speed of 0.5 mm/sec. The samples were cut with a rectangular shape and size of 5 mm × 25 mm × 1 mm. The samples were fixed to the machine by means of clamps.

### 2.4. Morphological Analysis by Scanning Electron Microscopy (SEM)

PVA and PVA composite scaffold morphology before and after chondrocytes seeding was investigated by SEM. Samples were fixed with 2.5% glutaraldehyde in 0.1 M cacodylate buffer (pH 7.2) for 24 h and then dehydrated with a graded ethanol series. After critical point drying and gold sputtering, they were observed by a scanning electron microscope (Stereoscan-205 S; Cambridge instruments, Pine Brook, NJ, USA).

### 2.5. Protein Quantitation Assay of Decellularized ECMs

Total ECM proteins were quantitated by bicinchoninic acid (BCA) method using the Pierce BCA Protein assay kit (Thermoscientific, Rockford, IL, USA) and following the manufacturer's instructions for protein detection on microplate wells. The analysis was performed on five different donor samples of W's J and AC matrix homogenates, obtained as described previously. Acetic acid homogenates (1 mL) were centrifuged at 12000 rpm for 5 min at 4°C and protein pellets were dissolved in 1 mL of 1% sodium dodecyl sulphate (SDS). The colorimetric reactions were analyzed at 562 nm using a Microplate autoreader EL 13 (BIO-TEK Instruments Inc., Winooski, Vermont, USA). The total protein amount was determined using a standard curve for bovine serum albumin (BSA).

### 2.6. Quality Assessment of ECM after Decellularization Treatment

For histological analysis, Wharton's jelly and articular cartilage fragments were soaked in cold isopentane and frozen in liquid nitrogen fumes and then kept at −80°C for 24 h. Samples were then ice-included and sliced in 7 *μ*m serial slices using a cryomicrotome (Leica CM 1850 UV). These sections were fixed with acetone and mounted with Vectashield mounting medium for fluorescence with DAPI (Vector Laboratories, Burlingame, CA, USA) to ascertain complete decellularization after each detergent-enzymatic cycle. In parallel, acellular samples were stained with Movat pentachromic and Masson trichromic kits to assess the maintenance of structural properties. As control, native W's J and AC samples were used.

### 2.7. Cartilage Harvest and Chondrocyte Isolation

Noncalcified human articular cartilage samples were collected from 3 donors who underwent total knee arthroplasty; only tissue from joints without signs of degenerative changes was used. The cartilage specimens were kept in basal medium DMEM and Nutrient Mixture F12, ratio 2 : 1, until further processed (within 24 h of sample collection). For chondrocyte isolation, cartilage was washed in PBS containing 2% of penicillin/streptomycin, minced finely, and digested with 0.1% collagenase B in basal medium at 37°C for 22 hours. The resulting cell suspension was collected and centrifuged at 1500 rpm for 5 min. Isolated cells were then seeded on 25 cm^2^ flasks (BD Falcon) at high density with complete medium as described below.

### 2.8. Chondrocyte Culture

Chondrocytes were cultured at 37°C in humidified atmosphere containing 5% CO_2_ with complete medium: DMEM/F12 (2 : 1) was added with 10% fetal bovine serum (FBS), 0.4 *μ*g/mL hydrocortisone, 8 ng/mL cholera toxin, 5 *μ*g/mL insulin, 24 *μ*g/mL adenine, 0.5 *μ*g/mL transferrin, 136 pg/mL triiodothyronine, and 1% penicillin/streptomycin solution. The medium was changed at the sixth day and then every 3-4 days.

### 2.9. Optical Microscopy Analysis

Cell cultures were daily observed by optical microscope DM/IL (Leica), and pictures were taken with a camera Nikon Digital Sight Ds-SMCc (Nikon Corporation).

### 2.10. RT-PCR

To investigate gene expression profile of chondrocyte primary cultures, mRNAs of specific cartilage markers were analysed using reverse transcription polymerase chain reaction (RT-PCR). Total RNA of cultured chondrocytes was first isolated using Trizol and quantified by NanoDrop 2000 (Thermo Fisher Scientific, Waltham, MA, USA) at 260 and 280 nm. Reverse transcription and specific amplification were performed in a single tube using QIAGEN OneStep RT-PCR Kit (Qiagen, Hilden, Germany, EU) according to the manufacturer's instructions. Specific oligoprimers (Life technologies, Carlsbad, CA, USA) designed on Gene Bank sequences (Table 1) were used and the expression of HPRT was considered as internal control. Finally, PCR products were separated by 7% polyacrylamide gel electrophoresis and visualized by silver nitrate staining. Pictures were taken using 3000 VersaDoc Gel Imaging System (Bio-Rad, Hercules, California, USA) and Quantity One software (Bio-Rad). Finally, band intensities were quantitated by densitometry, using* Image J* software.

### 2.11. Immunophenotype Characterization

Flow cytometry analysis was performed to identify chondrocyte specific immunophenotype. Cells were first harvested by treatment with trypsin-EDTA and resuspended in PBS and 0.2% BSA. Hence, chondrocytes were stained with phycoerythrin-conjugated antibodies, CD26, CD49c, CD44, and CD73; fluorescein isothiocyanate-conjugated antibodies, CD49e and CD151; and PerCP-Cyanine5-conjugated antibody, CD49f. Labeling occurred in 15 minutes at RT, in the dark. Isotypic antibodies served as controls. All the antibodies were purchased from BioLegend (San Diego, CA, USA), with the exception of CD151 and its isotype, purchased from Millipore (Billerica, MA, USA) (Table 2). For each sample, at least 10,000 events were analysed by a FACS Canto II cytometer (Becton Dickinson, Franklin Lakes, NJ, USA). Data were analysed by Flowing Software 2 and results were expressed as percentage of positive cells compared to the isotype negative control.

### 2.12. Chondrocyte Culture on Scaffolds

Primary human chondrocytes from passage 1, isolated and cultured as previously described, were used for seeding on scaffolds. PVA/W's J and PVA/AC scaffolds were washed 4 times of 2 h each in PBS solution containing 2% penicillin/streptomycin and then incubated at 37°C in basal medium overnight. Scaffolds were placed in a 24-well cell culture plate, seeded with chondrocytes (20,000 cells/cm^2^), and incubated at 37°C in a 5% CO_2_ humidified atmosphere.

### 2.13. Evaluation of Proliferative Activity

After 24 h and 7 and 14 days from seeding on scaffolds, cells were treated with 3-(4,5-dimethylthiazol-2-yl)-2,5-dimethyltetrazolium bromide (MTT) (0.5 mg/mL) for 4 h. Formazan precipitates were dissolved in 2-propanol acid (0.04 M HCl in 2-propanol) and optical density was measured at 570 nm, using a Microplate autoreader EL 13. Results were expressed as number of cells grown on seeded surface.

### 2.14. Statistical Analysis

We performed Student's *t*-test to determine the statistical significance of the data.

## 3. Results

### 3.1. Mechanical Properties of PVA Hydrogel

**Figure 1:**
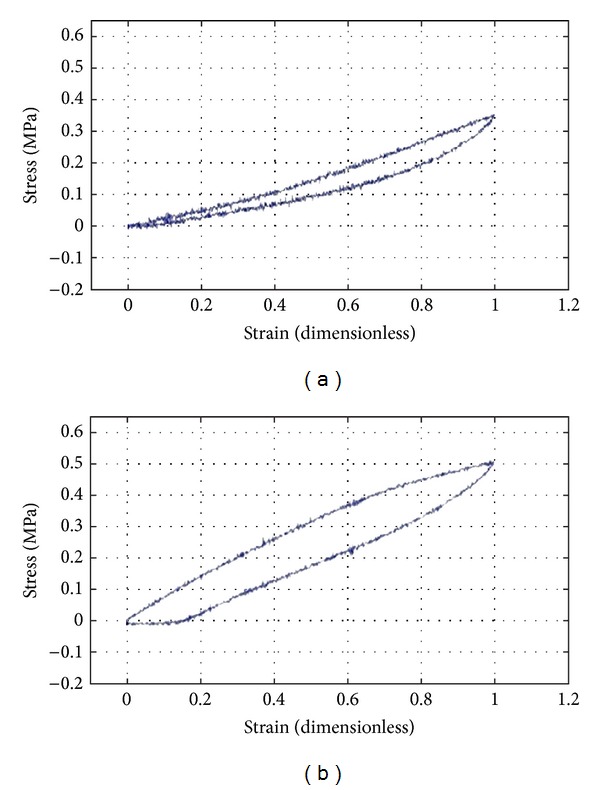
Stress-strain curves of PVA 16% (a) and PVA 25% (b) hydrogels.

Resilience is a measure of a material's ability to deform reversibly without loss of energy. To examine the possibility of using PVA as a mechanical support for cartilage regeneration, the resilience of 16% and 25% hydrogels was measured. Briefly, an electromechanical transducer exerted a traction force, stretching the specimen up to 100% of the initial length, while registering the applied strength. The stretching and relaxation curves of both biomaterials are represented by stress-strain profiles in Figure 1. Graphs show stress values relative to a 100% elongation and equal to 0.35 MPa for 16% PVA (Figure 1(a)) and 0.5 MPa for 25% PVA (Figure 1(b)).

### 3.2. Characterization of Scaffold Morphology (SEM)

**Figure 2:**
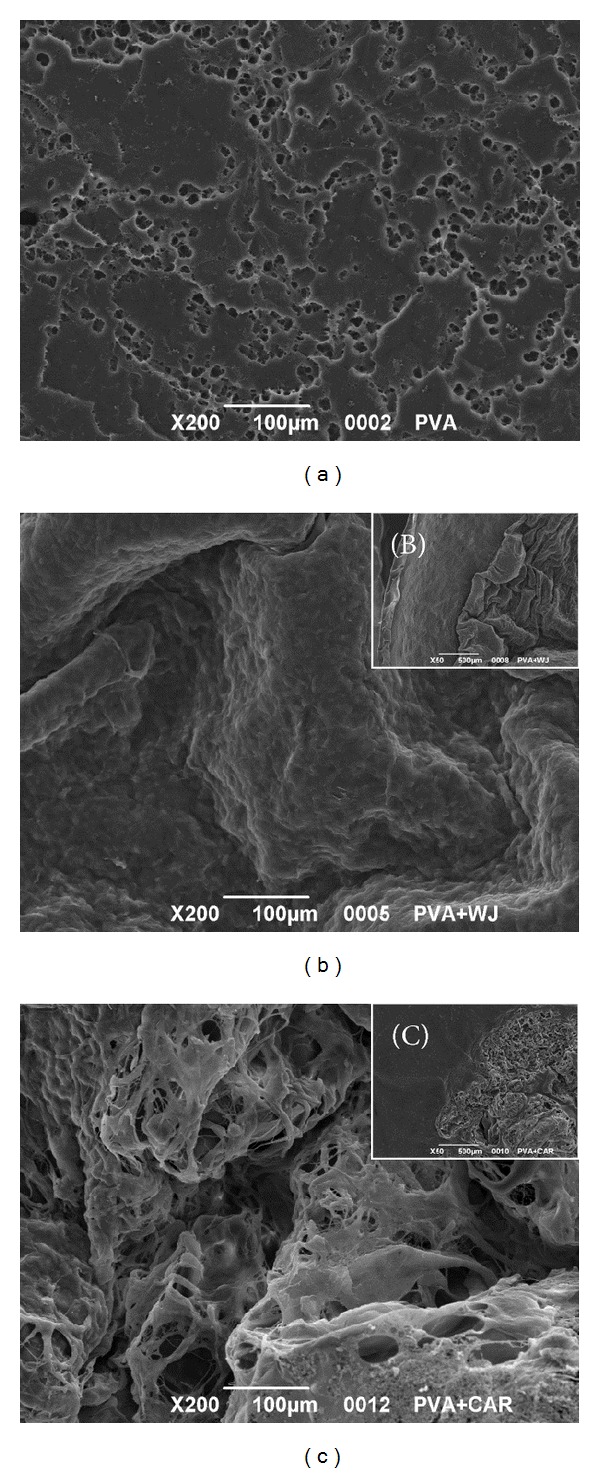
SEM investigation of PVA (a), PVA/W's J (b), and PVA/AC (c) scaffold surface morphology. The edge of PVA scaffold not covered by W's J and AC matrix is represented in (b) and (c), respectively. Magnification: ×200 (a, b, c); ×50 (b, c).

SEM micrographs were obtained to characterize the superficial morphology of scaffolds before chondrocyte seeding (Figure 2). PVA scaffolds showed a quite homogenous porous distribution with pore size ranging from 4 to 10 *μ*m (Figure 2(a)). PVA/W's J and PVA/AC scaffolds have a different surface morphology: the first is quite regular and smooth with convolution-like structures (Figure 2(b)); the second has a more irregular spongy appearance (Figure 2(c)).

### 3.3. Protein Quantitation of Decellularized ECMs

**Figure 3:**
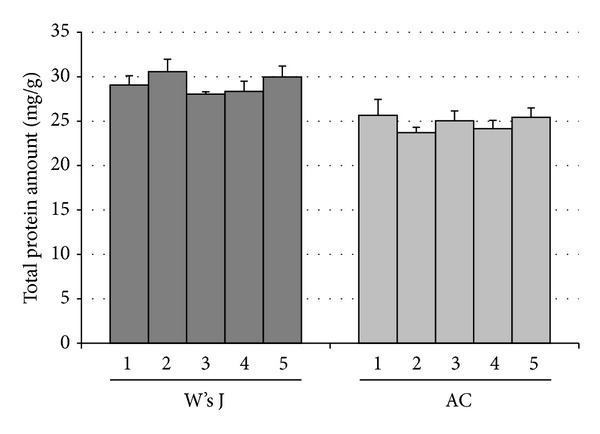
Total protein quantitation by BCA assay in decellularized Wharton's jelly and articular cartilage.

After ECM decellularization treatment, BCA assay was performed to control contingent sample-to-sample variations in total protein amount. Matrix homogenates of W's J and AC, gained from different donors, were compared. Total protein content for W's J and AC ECMs resulted in 29.2 and 24.8 mg per gram of tissue, respectively (mean values). No statistically significant difference was found between samples of each study group (Figure 3).

### 3.4. Evaluation of Acellular ECMs

**Figure 4:**
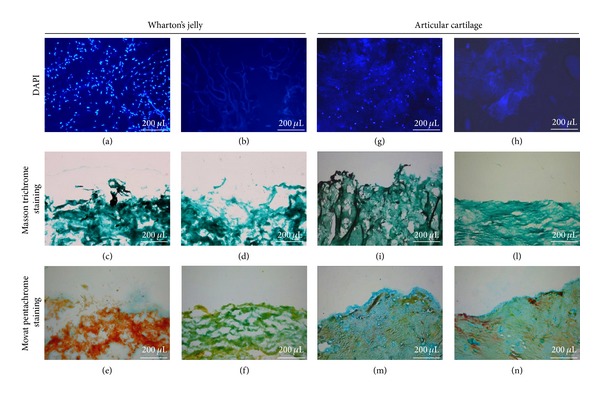
Histological evaluation of decellularized ECMs (b, d, f, h, l, and n) versus native tissues (a, c, e, g, i, and m). Magnification: ×100.

Umbilical cord Wharton's jelly and articular cartilage were completely decellularized with 3 and 7 detergent-enzymatic cycles, respectively; DAPI staining was used to assure decellularization degree after each cycle. Cartilage tissue resulted in more resistance to cell removal compared to Wharton's jelly; already one cycle induced an appreciable disappearance of cellular elements in the umbilical cord derived matrix. The histological sections of native and decellularized ECMs stained with DAPI are presented in Figures 4(a) and 4(g) and Figures 4(b) and 4(h), respectively.

ECMs morphology before and after the decellularization treatment was evaluated by means of Masson trichromic staining, which demonstrated a similar protein content of W's J and AC samples. In particular, both matrices mainly consist of collagen fibers and mucus, as shown by the green staining of native (Figures 4(c) and 4(i)) and decellularized (Figures 4(d) and 4(l)) tissues.

Movat pentachromic staining allowed us to detect red fibrin and yellow collagen components in native W's J (Figure 4(e)). Moreover, in native AC (Figure 4(m)) and acellular ECMs (Figures 4(f) and 4(n)), blue and yellow colors indicate the presence of mucins and collagen fibers, respectively.

### 3.5. Chondrocyte Monolayer Cultures

**Figure 5:**
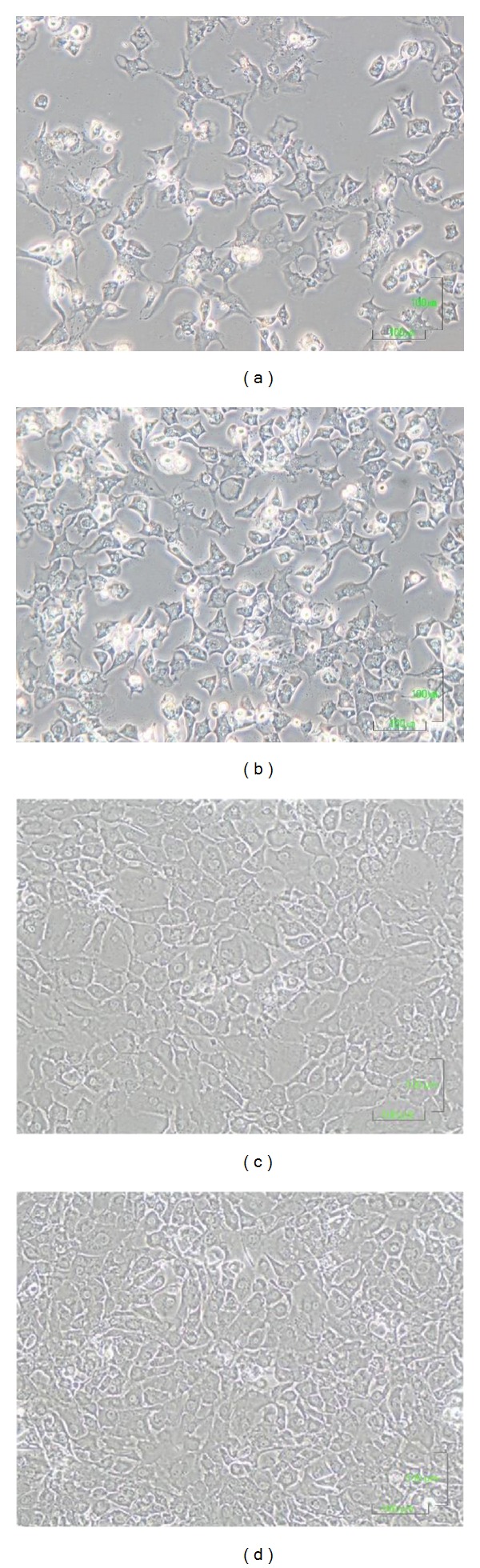
Morphological analysis by optical microscopy of human AC chondrocytes at passages 0 (a, c) and 4 (b, d) at a subconfluent (a, b) and confluent (c, d) state. Magnification: ×100.

Freshly isolated chondrocytes were small and round and they were initially grown as a suspension culture. Six days after AC enzymatic digestion, adherent cells were observed to spread across the flask and demonstrated clear boundaries and distinct nuclei (Figure 5(a)). In the subcultures, at a subconfluence state, chondrocytes showed the classic round or polygonal shape with small membrane extroflessions (Figure 5(b)). Once monolayer cultures reached 100% confluence, cells appeared to be smaller but maintained their characteristic morphology (Figures 5(c) and 5(d)). Chondrocytes were expanded in culture up to passage 4; hereafter, their proliferation rate started to decrease and their morphology changed to elongated fibroblast-like phenotype.

### 3.6. Characterization of Isolated Chondrocytes

**Figure 6:**
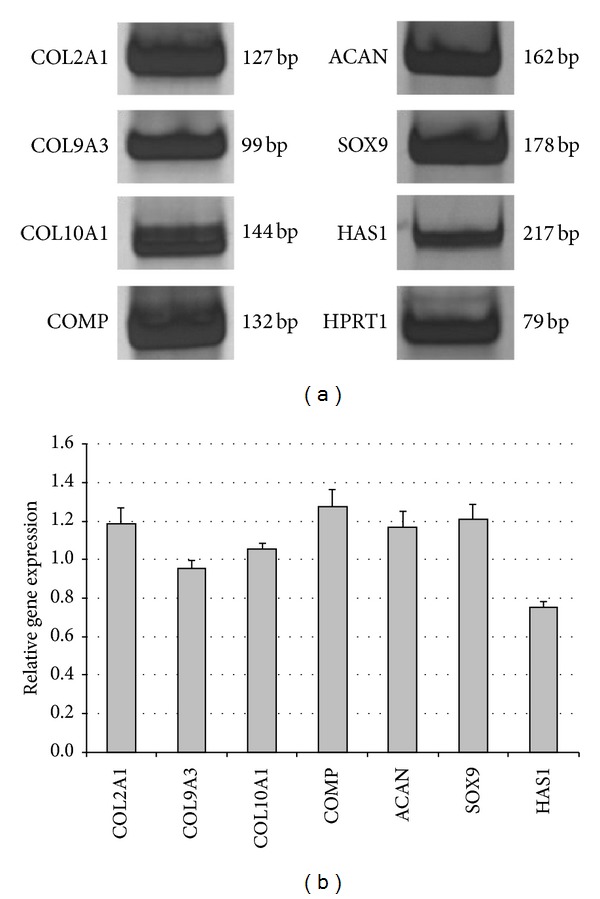
(a) Gene expression profile of isolated chondrocytes identified by RT-PCR. (b) Band intensities quantitation by densitometry. Relative expression of target genes is referred to HPRT1 expression.

Before seeding on 3D scaffolds, isolated human chondrocytes were characterized for the expression of specific cartilage markers. Gene expression analysis by RT-PCR showed that AC-derived cell populations are active in the transcription of typical chondrocyte mRNAs: collagen types II, IX, and X, cartilage oligomeric matrix protein, aggrecan, SOX9, and hyaluronan synthase (Figure 6(a)). As shown in Figure 6(b), densitometry quantitated band intensities were corrected for loading using housekeeping gene HPRT1 as a control and graphed as a ratio of HPRT1.

**Figure 7:**
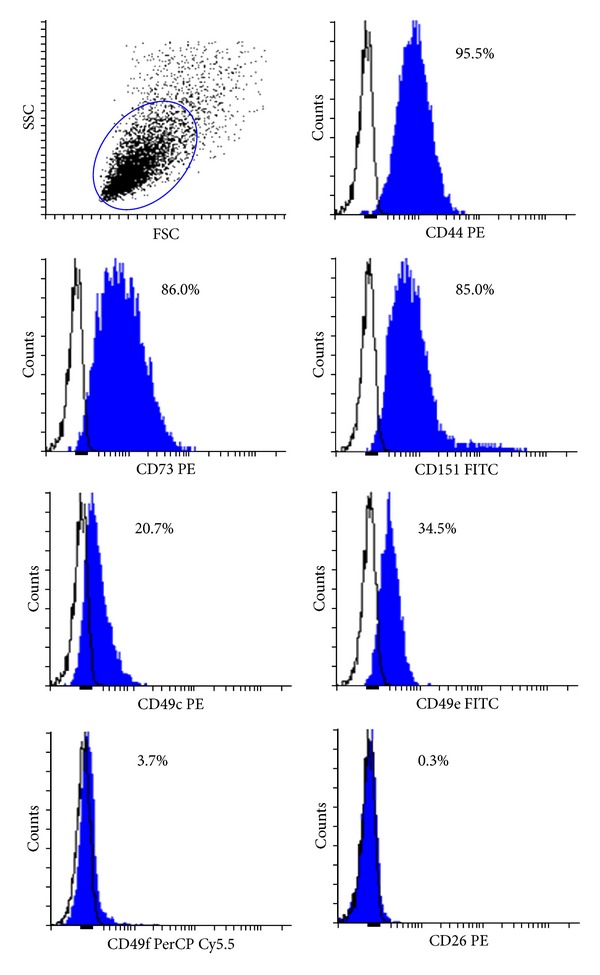
Immunophenotype evaluation of AC chondrocytes by flow cytometry. Data are expressed as percentage of positive cells (blue profile) compared to isotypic control (black profile).

To define the immunophenotype of AC chondrocytes, cell surface molecules expressed on cells obtained from 3 different donors (age range 32–85; mean 58.9) were evaluated by flow cytometry. Chondrocytes of each donor were cultured for 2 weeks in monolayer and passages 1 and 2 were investigated. The analysed cell surface molecules were classified into different categories according to their function: adhesion molecules (CD44; CD49c; CD49e; CD49f), receptors (CD151), and other surface molecules as ectoenzyme molecules (CD26; CD73). Chondrocytes subcultures were positive for CD44 (95.5%), CD73 (86.0%), CD151 (85.0%), CD49c (20.7%), and CD49e (34.5%); they showed low and negative expression of CD49f (3.7%) and CD26 (0.3%), respectively (Figure 7).

### 3.7. Chondrocytes Growth on 3D Scaffolds

Chondrocyte's distribution and proliferative activity on scaffolds were evaluated by SEM and MTT assay.

**Figure 8:**
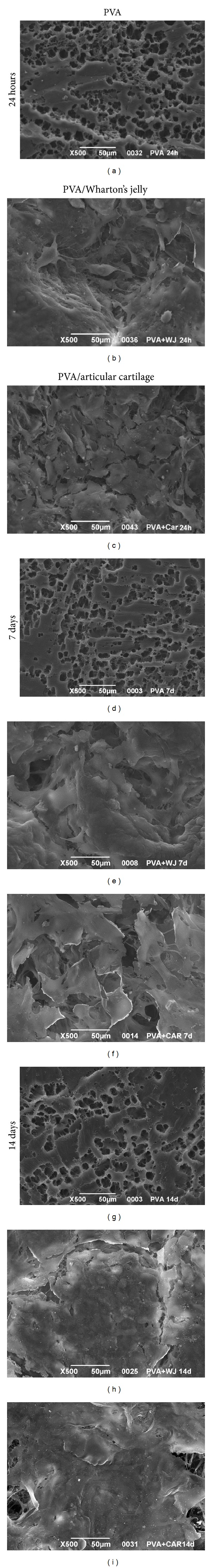
Evaluation of AC chondrocyte growth on 3D scaffolds by scanning electron microscopy. Cell cultures were analysed 24 h (a, b, c), 7 d (d, e, f), and 14 d (g, h, i) from seeding. Magnification: ×500.

According to SEM micrographs (Figure 8), on PVA scaffolds, any cell was visible since 24 h from seeding (Figure 8(a)); even at days 7 and 14 (Figures 8(d) and 8(g)), no cell adhesion and proliferation was observable. On the contrary, chondrocytes are visible both on PVA/W's J and on PVA/AC scaffolds. Twenty-four hours from seeding, on PVA/AC scaffolds, chondrocytes appeared well distributed with their typical round-shaped morphology (Figure 8(c)); cell organization on PVA/W's J scaffolds was less tidy (Figure 8(b)). At day 7, cells' limits on PVA/AC were still visible (Figure 8(e)), unlike ones of chondrocytes seeded on PVA/W's J scaffolds (Figure 8(f)). At day 14, chondrocytes extensively colonised both scaffold surfaces, forming a homogeneous monolayer (Figures 8(h) and 8(i)).

**Figure 9:**
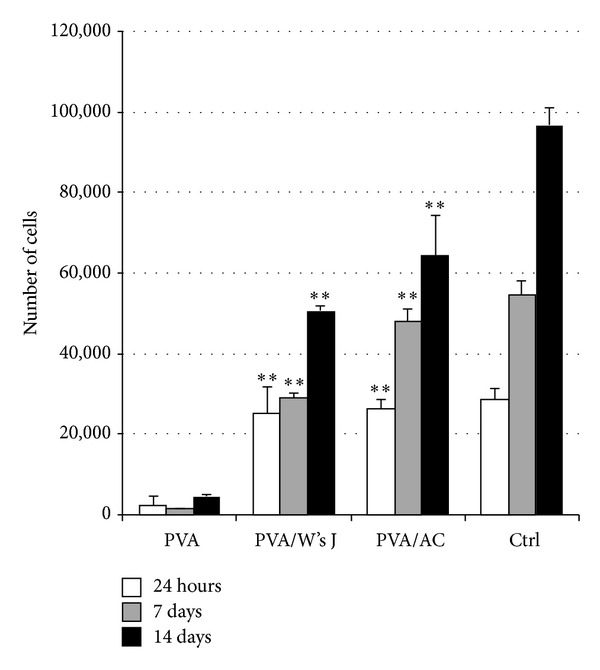
Cell proliferation following seeding on PVA, PVA/W's J, and PVA/AC scaffolds. Data are average of three independent experiments (**P ≤ 0.01: PVA/ECMs versus the corresponding PVA scaffold).

According to MTT assay (Figure 9), PVA did not sustain cell adhesion and proliferation, as previously demonstrated by SEM. Twenty-four hours from seeding, colonization of PVA/ECM scaffolds occurred, and cell number on composite supports was significantly higher (*P* ≤ 0.01) than that on native PVA. A progressive increase of cell number was observable from day 7 to day 14 on PVA/ECM scaffolds, where chondrocyte proliferation remained significantly higher (*P* ≤ 0.01) in comparison with PVA itself. Cell growth on tissue culture-treated polystyrene plates was considered as internal proliferation control (Ctrl).

## 4. Discussion

Articular hyaline cartilage is a soft tissue; it sustains the pressure between the hard ends of bones and it is subjected to particularly complex loads affecting its development and maintenance in the body [20, 21]. Because of its limited self-healing capacity, as it is an avascular and aneural tissue, even minor cartilage defects lead to mechanical joint instability and progressive damage [21, 22]. Cartilage damage is difficult to treat. Until now, many approaches have been investigated: arthroscopic repair procedures, soft tissue grafts, osteochondral transfer, autologous chondrocytes transplantation, and marrow stimulation [23], but average long-term results are unsatisfactory. A general drawback of these therapeutic strategies is that the newly formed tissue lacks the structural organization of cartilage; it has inferior mechanical properties compared to native tissue, and it is, therefore, prone to failure [21, 24]. Hence, the goal is to produce a repair tissue that has the same functional and mechanical properties of hyaline articular cartilage [25]. Cartilage restoration represents a challenge of musculoskeletal tissue engineering; despite that, the use of matrix scaffolds has paved the way for the use of functional tissue substitutes in the treatment of cartilage defects [22]. A wide range of natural and synthetic materials have been investigated as scaffolding for cartilage repair [26]. Natural scaffolds may face problems of immunogenic compatibility and batch inconsistency, while the properties offered by synthetic matrices provide much promise in the future of articular cartilage repair [25]. Amongst synthetic biomaterials, physically cross-linked PVA hydrogels become suitable for soft tissue applications: thanks to their biocompatibility and mechanical properties, they have been proposed for many biomedical applications, even as cartilage substitutes [15]. Mechanical properties of the gel can be modulated acting on different variables: polymer molecular weight, number of freezing/thawing cycles, and polymer solution concentration [27]. Varying the polymer wt%, we realized two different PVA hydrogels (PVA 16 wt% versus PVA 25 wt%), which were tested for their tensile strength. PVA 16 wt% hydrogel is more elastic than 25 wt% one. As proved by stress-strain profiles presented, it did not maintain the residual strain when subjected to tensile strength, revealing high elasticity. However, cell adherence on PVA hydrogels is inhibited by its highly hydrophilic nature [28]. Many authors demonstrated ECM-based scaffold efficacy in creating a more suitable microenvironment to sustain cellular adhesion. Extracellular matrix is a reservoir of structural and functional proteins like collagens, glycoproteins, proteoglycans, mucins, and elastic fibres as well as a known repository for a variety of growth factors. As* in vivo* it is progressively degraded by proteinases, it can result in the exposure of new recognition sites with potent bioactivity [29]. In this work, we decided to combine PVA mechanical properties with ECM features. Our aim was to provide a supportive biomimetic microenvironment for chondrocytes to produce articular cartilage, taking advantage of both PVA and ECM. In particular, we considered an alternative matrix source: we focused our attention on a new ECM represented by decellularized Wharton's jelly, in comparison with decellularized cartilage matrix.

The research of a new biological ECM useful in cartilage restoration arises from the need to identify an easily available resource suitable in sustaining chondrocytes adhesion and proliferation, even if not specific. Every tissue and organ contains an ECM with unique composition that consists of the secreted products of resident cells [29]. The main components of Wharton's jelly were ECM proteins such as collagen and fibronectin. Previous studies demonstrated that Wharton's jelly contains growth factors such as insulin-like growth factor I (IGF-1), fibroblast growth factor (FGF), transforming growth factor *β* (TGF*β*), platelet-derived growth factor (PDGF), epidermal growth factor (EGF), and ECM proteins [30]. These peptides and growth factors induce Wharton's jelly cells to produce large amounts of collagen and glycosaminoglycans [31], also typical components of cartilage matrix [32]. The aim of decellularization treatment is to decrease the antigenicity of matrices, through an efficient removal of cellular and nuclear material, preserving its composition [33]. Histological analysis of decellularized Wharton's jelly and articular cartilage ECM demonstrated the effectiveness of the treatment. A different number of detergent-enzymatic cycles, 3 and 7 cycles, respectively, were performed. According to DAPI staining, native Wharton's jelly showed a higher cellular density in comparison with native articular cartilage; nevertheless, its complete decellularization was easier to achieve. This may be related to a different tissue macroscopic aspect: while chondrocytes are deeply embedded in matrix, Wharton's jelly permits a better exposure of its cellular elements to sodium deoxycholate and DNase-I, as well as to the osmotic effect of deionized water. To control and quantify W's J and AC batch-to-batch variations, decellularized ECMs were analysed in regard to their total protein levels. Matrix homogenates, gained from different donors, showed a similar profile to BCA assay: no significant difference was detected between samples of the same group. According to this data, sample-to-sample variations are negligible. Extracellular matrix characterization before and after detergent-enzymatic treatment was also achieved by means of Masson trichrome and Movat pentachrome staining. According to Masson trichrome, both Wharton's jelly and articular cartilage maintain their collagen and mucus content (deeply green appearance). Movat pentachrome staining confirmed the concomitant presence of collagen and mucus, even after the treatment. The resulting green leading colour is due to the overlapping between yellow (referred to collagen and reticular fibres) and blue (referred to mucus). However, the detergent-enzymatic treatment seemed to remove or reduce fibrinoid elements' expression. This ECMs characterization highlighted a similar histomorphology for Wharton's jelly and cartilage, supporting our theory.

The chief aim of many authors is to preserve tissue or organ histoarchitecture from a too aggressive decellularization treatment; on the contrary, we approached ECMs in a different manner. We take advantage of matrices macromolecules instead of their superstructure. ECM homogenates are an interesting and innovative manner of working with matrices. Choosing an adequate mould and modulating the needed quantity, the liquid suspension obtained can be used to create tailored scaffolds. Furthermore, the lyophilization process they subsequently undergo makes them easy to store. The two different lyophilized matrices realized were examined by SEM for their fine structure: the cartilage derived one appeared spongier than the Wharton's jelly analogue.

Physical cross-linking of lyophilized matrices with PVA solutions led to three-dimensional composite scaffolds. As chondrocytes usually tend to dedifferentiate to fibroblasts when grown in a monolayer culture, a three-dimensional culture system can be used to maintain the chondrogenic phenotype [34]. Before seeding on scaffolds, we isolated cells from human articular cartilage; we confirmed their chondrocyte gene expression profile and phenotype through RT-PCR and flow cytometry analysis.

The viscoelastic properties of articular cartilage arise from the composition of its ECM, which consists primarily of type II collagen but also of collagen types IX and X and a proteoglycan termed aggrecan (ACAN) [35]. Aggrecan is retained in cartilage by binding to long filaments of another glycosaminoglycan, hyaluronan (HA), which is synthesized at the plasma membrane level by an enzyme called hyaluronan synthase (HAS) [36]. Moreover, one of the major noncollagenous proteins in the cartilage is COMP, which represents a useful marker of differentiation state of primary chondrocytes [37]. The synthesis of this cartilage-specific ECM requires the expression of genes associated with the specific chondrocyte phenotype, controlled by the transcription factor SOX9 [35]. According to RT-PCR analysis, cells isolated for this study express specific cartilage markers at the mRNA level, showing a gene expression profile typical of articular chondrocytes.

Expanded chondrocytes were, thus, assessed by flow cytometry. We purchased antibodies against several CDs, typically used to characterize the phenotype of mesenchymal progenitor cells [38, 39] and recently introduced to determine the stage of differentiation of human articular chondrocytes [39, 40]. In this study, we confirmed the previously reported expression of several articular chondrocyte surface markers: the hyaluronan receptor CD44, the ectoenzyme CD73, the integrins *α*3 (CD49c), *α*5 (CD49e), and the tetraspanin CD151 [39, 40]. According to Grogan and colleagues [39], chondrocytes with marked chondrogenic capacity express high levels of the hyaluronan receptor CD44, the *α*3 integrin subunit CD49c, and the tetraspanin CD151. They are surface molecules involved in the early stages of cartilage development; all of them were present in the chondrocytes we investigated. Moreover, these proteins are responsible for establishing cell-cell and cell-matrix interactions. These processes are known to be important mediators of mesenchymal condensation, which is in turn necessary for initiation of chondrogenesis [41]. Hence, high expression levels of these membrane proteins might increase the propensity of the cells to differentiate and produce cartilage ECM. Markers characteristics of mesenchymal progenitor cells, that is, CD44 and CD73 [38], have been shown to be expressed in high-chondrogenic-capacity populations [39]. This suggests that, within a chondrocyte culture, subpopulations with higher capacity to form cartilage might correspond to those with progenitor characteristics.

After characterization of scaffold histomorphology and chondrocyte gene expression profile and specific immunophenotype, we seeded a known cell amount of 20,000 cells/cm^2^ on PVA, PVA/W's J, and PVA/AC supports. We evaluated chondrocyte adhesion and proliferation at three different end-points: 24 h and 7 and 14 days. If PVA itself clearly demonstrated its absolute inability to sustain chondrocyte proliferation, cells on composite scaffolds revealed a progressive increasing growth trend. At 24 h from seeding, cells adhered on PVA/ECMs, which were able to sustain cell proliferation up to the last end-point considered (14 days). According to SEM micrographs, chondrocytes on PVA/AC showed a more specific morphology and a more tidy orientation on the scaffold surface. In parallel, PVA/W's J revealed a singular attitude to sustain cell proliferation despite its aspecific origin. Hence, as stressed also by MTT proliferation assay, our* in vitro* model confirmed the starting hypothesis regarding the possibility to use Wharton's jelly in composite scaffolds that mimic articular cartilage.

## 5. Conclusions

Decellularized Wharton's jelly matrix is an attractive reservoir of macromolecules. Our preliminary results proved that it promotes chondrocyte adhesion, representing an idoneous biomimetic microenvironment despite its aspecific nature. Further investigations are necessary to evaluate phenotype maintenance of chondrocytes grown upon PVA/W's J scaffolds. As a future goal, these composite supports will be tested* in vivo* using rabbit models of articular joint defects.

## Figures and Tables

**Table 1:** Primers for RT-PCR.

Gene	Forward primer 5′→3′ Reverse primer 3′→5′	GenBank accession	Base pair (bp)
Collagen, type II, alpha 1 (COL2A1)	F: CCGGGCAGAGGGCAATAGCAGGTT R: CAATGATGGGGAGGCGTGAG	NM_001844.4	127
Collagen, type IX, alpha 3 (COL9A3)	F: AATCAGGCTCTCGAAGCTCATAAAA R: CCTGCCACACCCCCGCTCCTTCAT	NM_001853.3	99
Collagen, type X, alpha 1 (COL10A1)	F: GAACTCCCAGCACGCAGAATCC R: GTGTTGGGTAGTGGGCCTTTTATG	NM_000493.3	144
Cartilage oligomeric matrix protein (COMP)	F: CCGGAGGGTGACGCGCAGATTGA R: TGCCCTCGAAGTCCACGCCATTGAA	NM_000095.2	132
Aggrecan (ACAN)	F: GGCTGCTGTCCCCGTAGAAGA R: GGGAGGCCAAGTAGGAAGGAT	NM_001135.3	162
Transcription factor SOX9 (SOX9)	F: CTGGGCAAGCTCTGGAGA R: ATGTGCGTCTGCTCCGTG	NM_000346.3	178
Hyaluronan synthase 1 (HAS1)	F: CAGACCCACTGCGATGAGAC R: CCACCAGGTGCGCTGAAA	NM_001523.2	217
Hypoxanthine phosphoribosyltransferase 1 (HPRT1)	F: ATGGACAGGACTGAACGTCTTGCT R: TTGAGCACACAGAGGGCTACAATG	NM_000194.2	79

**Table 2:** Antibodies used for flow cytometry.

Antigen recognized	Isotype	Fluorochrome	Category
CD26 (peptidase IV)	IgG2a	PE	Ectoenzyme
CD44	IgG1	PE	Adhesion molecule
CD49c (*α*3 integrin chain)	IgG1	PE	Adhesion molecule
CD49e (*α*5 integrin chain)	IgG2b	FITC	Adhesion molecule
CD49f (*α*6 integrin chain)	IgG2a	PerCP/Cy5.5	Adhesion molecule
CD73 (5′-nucleotidase)	IgG1	PE	Ectoenzyme
CD151	IgG1	FITC	Tetraspanin
